# The complete chloroplast genome of *Solanum melongena* ‘Yunqie 9’

**DOI:** 10.1080/23802359.2024.2438290

**Published:** 2025-01-12

**Authors:** Mao Sun, Yanan Yu, Yulei Yang, Yaju Gong, Min Gui, Xiangshu Dong, Liyan Wu, Guanghui Du

**Affiliations:** a School of Agriculture, Yunnan University, Kunming, China; b Horticultural Research Institute, Yunnan Academy of Agricultural Sciences, Kunming, China

**Keywords:** *Solanum melongena*, chloroplast genome, phylogenetic analysis

## Abstract

*Solanum melongena* ‘Yunqie 9’ was selected by the Horticultural Research Institute of Yunnan Academy of Agricultural Sciences based on the local environment of Yunnan Province. It is excellent in fruit quality and yield, but it is relatively weak in disease resistance. No information on complete chloroplast genome and position in the phylogeny of *Solanum* to restrict its genetic improvement. In this study, the chloroplast genome of ‘Yunqie 9’ was sequenced using Illumina high-throughput sequencing technology. The size of the complete chloroplast genome was 155,579 bp in length with an GC content of 37.70%, composed of a large single-copy region (86,189 bp), a small single-copy region (18,504 bp), and a pair of inverted repeat regions (25,443 bp). A total of 134 coding genes were annotated in the entire chloroplast genome, including 89 protein-coding genes, 37 transfer RNA genes, and 8 ribosomal RNA genes. Phylogenetic analysis based on chloroplast genome sequences of 20 species in the Solanaceae family indicated that except for the cultivated *Solanum*, ’Yunqie 9’ was closely related to *Solanum melongena*×*Solanum torvum* and *Solanum insanum*.

## 1. Introduction

**Figure 1.**
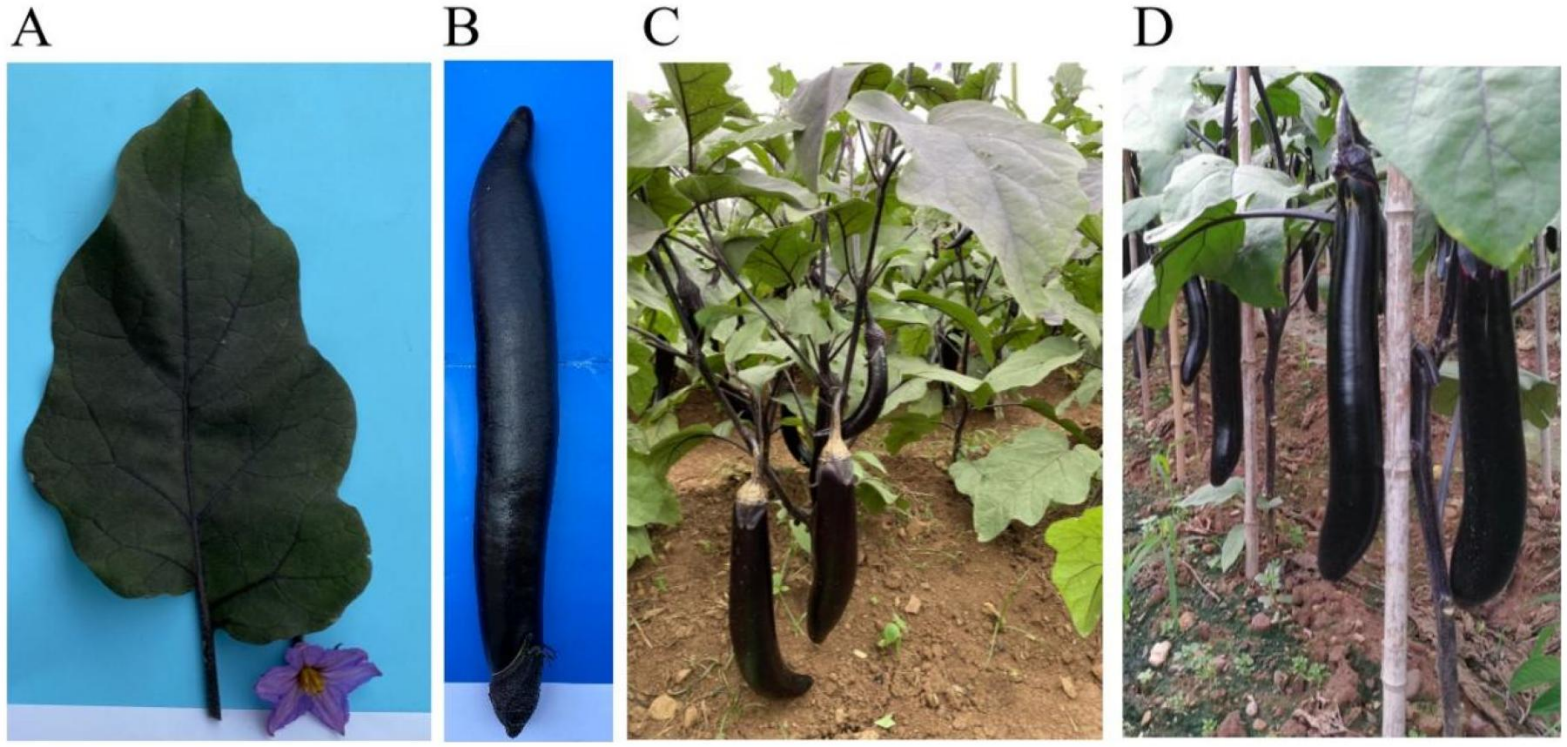
The photographs of *Solanum melongena* ‘Yunqie 9’. (A) Flower (light purple color) and leaf (oval-shaped, with light purple leaf vein, and no leaf thorns); (B) Fruit (dark purple color, long cylindrical shape, and with purple calyx); (C,D) Eggplant plants in the field (upright plant habit). (these photos were taken by the corresponding author, professor Liyan Wu, without any copyright issues).

Eggplant (*Solanum melongena* Linnaeus. 1753, 2*n* = 2*x* = 24), an annual plant belonging to the Solanaceae family, originates from tropical Southeast Asia. It is one of the important vegetable crops in the world. Eggplant fruit is rich in solanine, vitamin P, anthocyanin, and other nutrients, which has high nutritional and medicinal value (San José et al. 2014). Yunnan Province is located in the Yunnan-Guizhou Plateau, with low latitude, high altitude, cool climate, abundant sunlight, distinct wet and dry seasons, and large diurnal temperature difference in summer and autumn, has the natural advantages of developing high-quality vegetables (Wang et al. 2018). *Solanum melongena* ‘Yunqie 9′, bred by the Horticultural Research Institute of Yunnan Academy of Agricultural Sciences in 2020, has long, glossy fruits with an average length of 32 cm (Figure 1). The fruit of ‘Yunqie 9′ has green and white flesh, with an average yield of about 5370 kg per 667 m^2^. ‘Yunqie 9′ is one of the new eggplant varieties in Yunnan Province with independent intellectual property rights and suitable for local cultivation. ‘Yunqie 9′ showed vigorous growth, good in fruit shape, excellent fruit quality and high yield, but it is relatively weak in disease resistance. Chloroplast genome is a relatively independent genetic system outside the nucleus, with a set of genes related to photosynthesis, energy metabolism, protein synthesis, and nitrogen and sulfur assimilation (Pottosin and Shabala 2016). The advantages of chloroplast genome are small in size, stable genetic traits, maternal inheritance, and can be easily sequenced, therefore are widely used in plant species identification, phylogeny and genetic analysis (Twyford and Ness 2017; Zhang et al. 2019). However, the complete chloroplast sequence of ‘Yunqie 9′ and its phylogenetic position in Solanaceae are still unknow. This study combined second-generation high-throughput sequencing and third-generation sequencing technology to obtain the full length chloroplast genome sequence of the new eggplant variety ‘Yunqie 9′ and compared it with the chloroplast genome of several other species (or resources) in the Solanaceae family, to reveal the evolutionary relationship of ‘Yunqie 9′ and its position in the phylogeny of *Solanum*. It will be helpful to find suitable cross breeding materials and grafting rootstocks of ‘Yunqie 9′, and to improve its disease resistance ability.

## 2. Materials and methods

In this study, fresh leaves of ‘Yunqie 9′ were collected from the greenhouse of the Horticultural Research Institute of Yunnan Academy of Agricultural Sciences (25.12°N, 102.76°E, 1954 m), Kunming, China. A specimen was deposited at Horticultural Research Institute of Yunnan Academy of Agricultural Sciences (http://www.ynhort.cn, Qiuyue Zhong, yyskgk@yaas.org.cn) under the voucher number SM000009. A modified CTAB method was used to extract high-quality total genomic DNA (Yang et al. 2014). The quality and quantity of the extracted DNA were examined using a NanoDrop 2000 spectrophotometer (NanoDrop Technologies, Wilmington, DE, USA), Qubit dsDNA HS Assay Kit on a Qubit 3.0 Fluorometer (Life Technologies, Carlsbad, CA, USA) and electrophoresis on a 0.8% agarose gel The sequencing coverage across the chloroplast gemone of ‘Yunqie 9′ was showed in Supplemental Figure S1. Then, the qualified genomic DNA (Supplemental Table S1) was sent to Shanghai Majorbio Biopharm Technology Company (Shanghai, China) for sequencing by Illumina NovaSeq. Raw reads were filtered by using the NGSQC toolkit with default parameters to obtain clean reads of high quality (Patel and Jain 2012). The clean reads were trimmed and assembled by NOVOPlasty software (Dierckxsens et al. 2017). Then, the assembled sequences were analyzed for possible assembly errors by collinearity with related species using Mummer (http://mummer.sourceforge.net/manual/). Finally, the gene structure (protein coding genes, tRNA and rRNA) of assembled chloroplast genome was annotated by PGA program (Qu et al. 2019).

The phylogenetic tree was constructed using a maximum likelihood method with a Kimura 2-parameter model based on chloroplast coding sequences. This phylogeny analysis was conducted using MEGA 6.0 with 1,000 bootstrapping (Tamura et al. 2013).

## 3. Results

**Figure 2.**
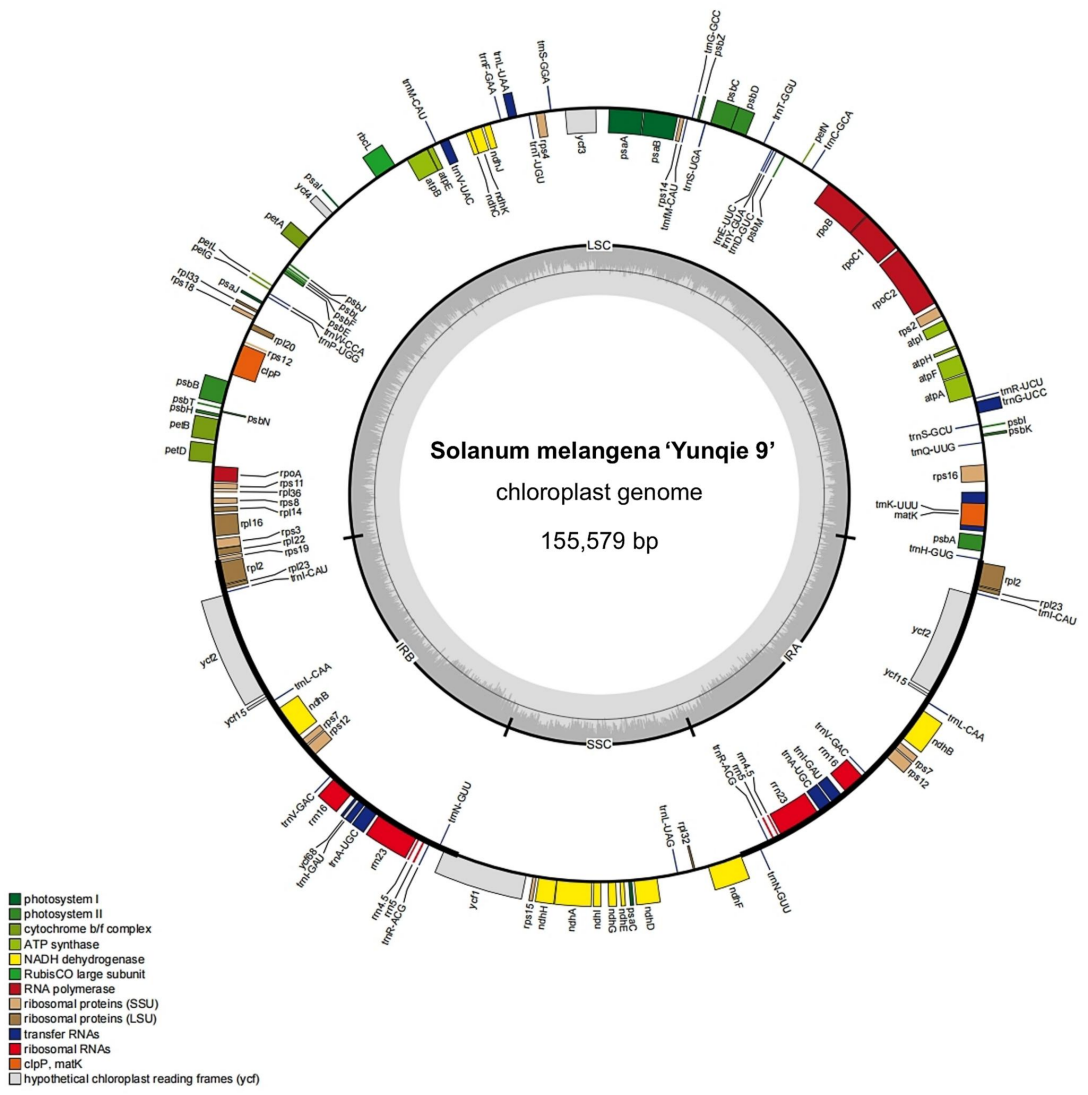
Structure of chloroplast genome of ‘Yunqie 9’. Genes with different functions are shown in different colors. Genes within circles are transcribed clockwise and genes outside circles are transcribed counterclockwise. LSC: large single-copy region; SSC: small single-copy region; IR: inverted repeat.

The original sequencing data was matched to the assembled chloroplast genome, and the coverage depth of the data at each base on the genome was counted. In this project, the average sequencing depth of the chloroplast genome was as high as 2186X, which ensured the accuracy of the assembly results (Supplemental Figure S1). The size of the complete chloroplast genome of ‘Yunqie 9′ (GenBank accession number: OP688482) was 155,579 bp, and its overall GC content was 37.7%. The chloroplast genome had a characteristic quadripartite circular structure, and composed of a large single-copy region (86,189 bp), a small single-copy region (18,504 bp), and a pair of inverted repeat regions (25,443 bp). In addition, there were 89 protein-coding genes, 37 transfer RNA (tRNA) genes, and 8 ribosomal RNA (rRNA) genes in the entire genome (Figure 2). The chloroplast genome included 11 cis-splicing genes (*ndhB, rpl2, rps16*, *atpF*, *rpoC1*, *ycf3*, *clpP*, *petB*, *petD*, *rpl16,* and *ndhA*). Two of these genes (*ndhB* and *rpl2*) were duplicated and one appeared as a trans-spliced gene. The structures of cis-splicing and trans-splicing genes are shown in Supplemental Figures S2 and S3.

**Figure 3.**
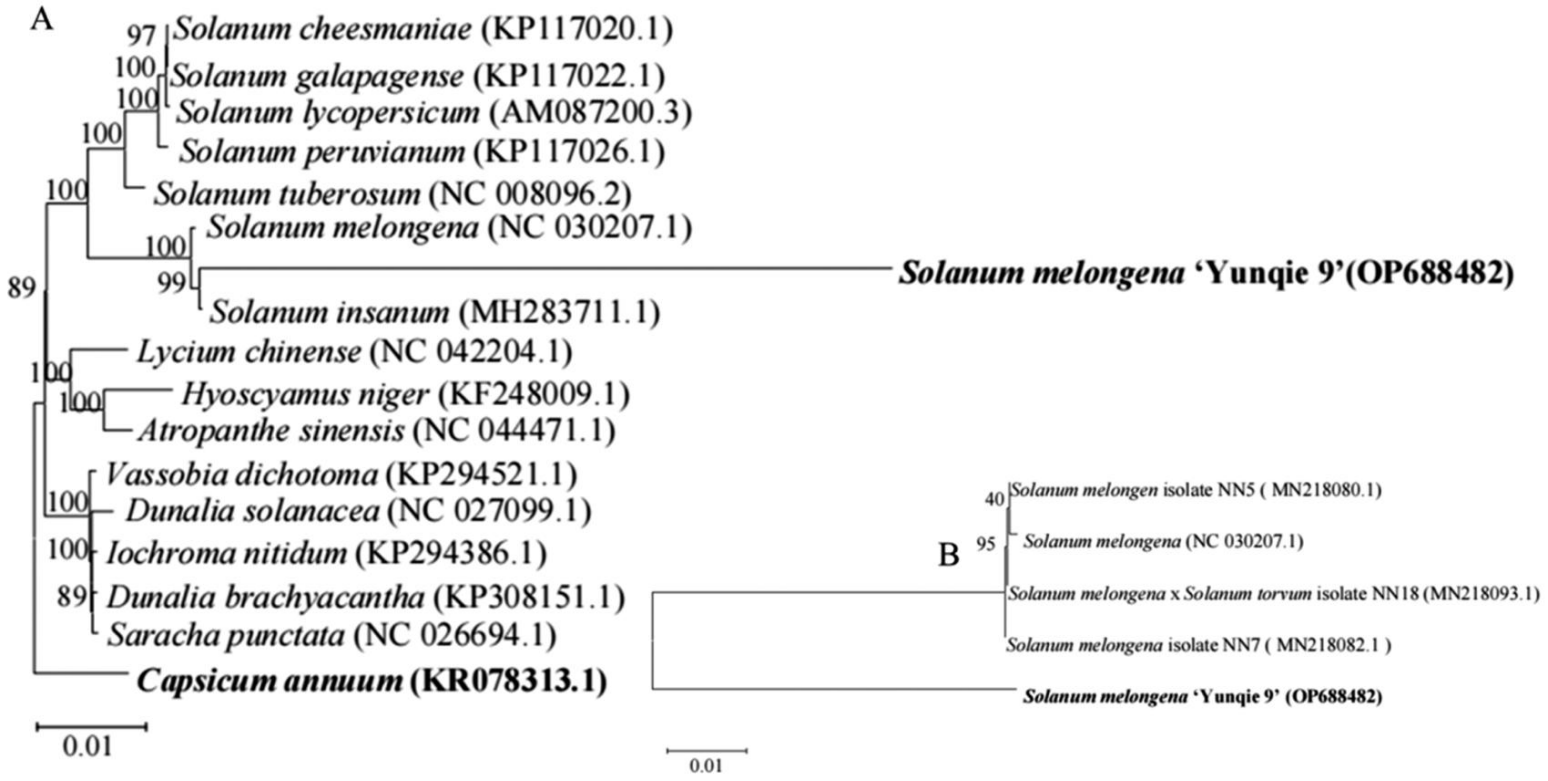
Phylogenetic tree of 21 complete chloroplast genomes in Solanaceae species (Figure a shows the phylogeny of different *Solanaceae* species, and Figure B shows the phylogenetic map of different eggplant varieties). The phylogenetic tree was constructed using a maximum likelihood method with a Kimura 2-parameter model based on chloroplast coding sequences. Numbers at each node indicate the bootstrap support values. *Capsicum annuum* was served as outgroup. Chloroplast genome accession number was used in this phylogeny analysis, *Solanum melongena* ‘Yunqie 9’ OP688482 (it is the target chloroplast sequence of this study), *Solanum cheesmaniae* KP117020.1, *Solanum galapagense* KP117022.1, *Solanum lycopersicum* AM087200.3 (Kahlau et al. 2006), *Solanum peruvianum* KP117026.1, *Solanum bulbocastanum* DQ347958.1 (Zhang et al. 2021), *Solanum commersonii* KM489054.2, *Solanm tuberosum* NC 008096.2, *Solanum melongena* NC 030207.1 *Solanum melongena* MN218082.1, *Solanum melongena* MN218080.1 (Zhang et al. 2024), *Solanuum melongena* × *Solanum torvum* MN218093.1, *Hyoscyamus niger* KF248009.1 (Sanchez-Puerta and Abbona 2014), *Lycium chinense* NC 042204.1, *Vassobia dichotoma* KP294521.1, *Dunalia solanacea* NC 027099.1, *Iochroma nitidum* KP294386.1, *Dunalia brachyacantha* KP308151.1, *Saracha punctata* NC 026694.1, *Capsicum amnum* KR078313 (Magdy et al. 2019).

In order to determine the taxonomic and evolutionary status of ‘Yunqie 9′ in the Solanaceae family, the chloroplast genome sequences of 21 species were selected for chloroplast genome sequence alignment using MAFFT software (Kumar et al. 2016). These 21 published sequences were obtained from NCBI GenBank, these included 10 important Solanum species and other 9 important Solanaceae species, and *Capsicum annuum* (KR078313.1) were selected as an outgroup (Figure 3). The results showed that ‘Yunqie 9′ was closely related to *Solanum insanum* (MH283711.1), *Solanum melongena* (MN218082.1), *Solanum melongena* (MN218080.1), and *Solanum melongena*×*Solanum torvum* (MN218093.1), reported by Zhang et al. (2021), Aubriot et al. (2018), and Ranil et al. (2017).

## 4. Discussion and conclusions

In this study, the complete chloroplast genome size of ‘Yunqie 9′ was 155,579 bp and the total GC content was 37.7%, it was high similarity with other species in *Solanum*, such as *Solanum melongena* (Ding et al. 2016), *Solanum iopetalum* (Park 2023), *Solanum hougasii* (Park 2022), and *Solanum chacoense* (Cho et al. 2017). This indicated that the chloroplast genome of ‘Yunqie 9′ may be highly conserved and stable evolution.

The complete chloroplast genome of *Solanum melongena* ‘Yunqie 9′ reported in this study provided basic genetic data and showed the phylogenetic relationships with 20 species to confirm that the plastome genome of the target species is closely related to the *Solanum melongena*×*Solanum torvum* and *Solanum insanum. Solanum insanum* is a member of the *Solanum* clade (Weese and Bohs 2010; Vorontsova et al. 2013), which is a part of the Old World clade of the spiny solanums (Levin et al. 2006). It is probably the wild progenitor of the cultivated eggplant (*Solanum melongena*). Research showed that *Solanum insanum* could adapt to drought, resist various diseases, and have a strong adaptability to different environmental conditions (Aubriot et al. 2016). So whether *Solanum insanum* can cross with ‘Yunqie 9′ or it can be used as the grafting stock of ‘Yunqie 9′ to produce high yield and good resistance varieties, it needs to be further verified.

These results will provide a useful resource for conservation, genetics and will provide reference for future studies on the chloroplasts of Solanaceae species.

## Supplementary Material

Supplementary document .docx

## Data Availability

The genome sequence data that support the findings of this study are openly available in GenBank of NCBI at https://www.ncbi.nlm.nih.gov, reference number OP688482.1. The associated BioProject, SRA, and BioSample numbers are PRJNA1024941, SRR26304029, and SAMN37705625, respectively.
