# Supplementary material for: The complete chloroplast genome of Solanum melongena ‘Yunqie 9’
Source: Mitochondrial DNA B Resour. 2025 Jan 12;10(2):103–7. doi: 10.1080/23802359.2024.2438290 (PMC11730860; doi:10.1080/23802359.2024.2438290)
Supplement: Supplementary document .docx [file TMDN_A_2438290_SM0281.docx]

**Table S1** Chloroplast DNA quality data of leaves in ‘Yunqie 9’

| Sample  Name | Sample  type | DNA  concentration  (ng/μl) | Total DNA amount  (μg) | OD260/280 | OD260/230 |
| --- | --- | --- | --- | --- | --- |
| Yunqie 9 | Leaf | 154.00 | 12.32 | 2.09 | 2.25 |





**Figure S1** Chloroplast gemone coverage of ‘Yunqie 9’. The map of sequencing depth and coverage, representing the sequencing depth across the chloroplast genome. The red line indicates the sequencing depth at each genomic position, while the blue dashed line represents the average sequencing depth of 2186.36X.


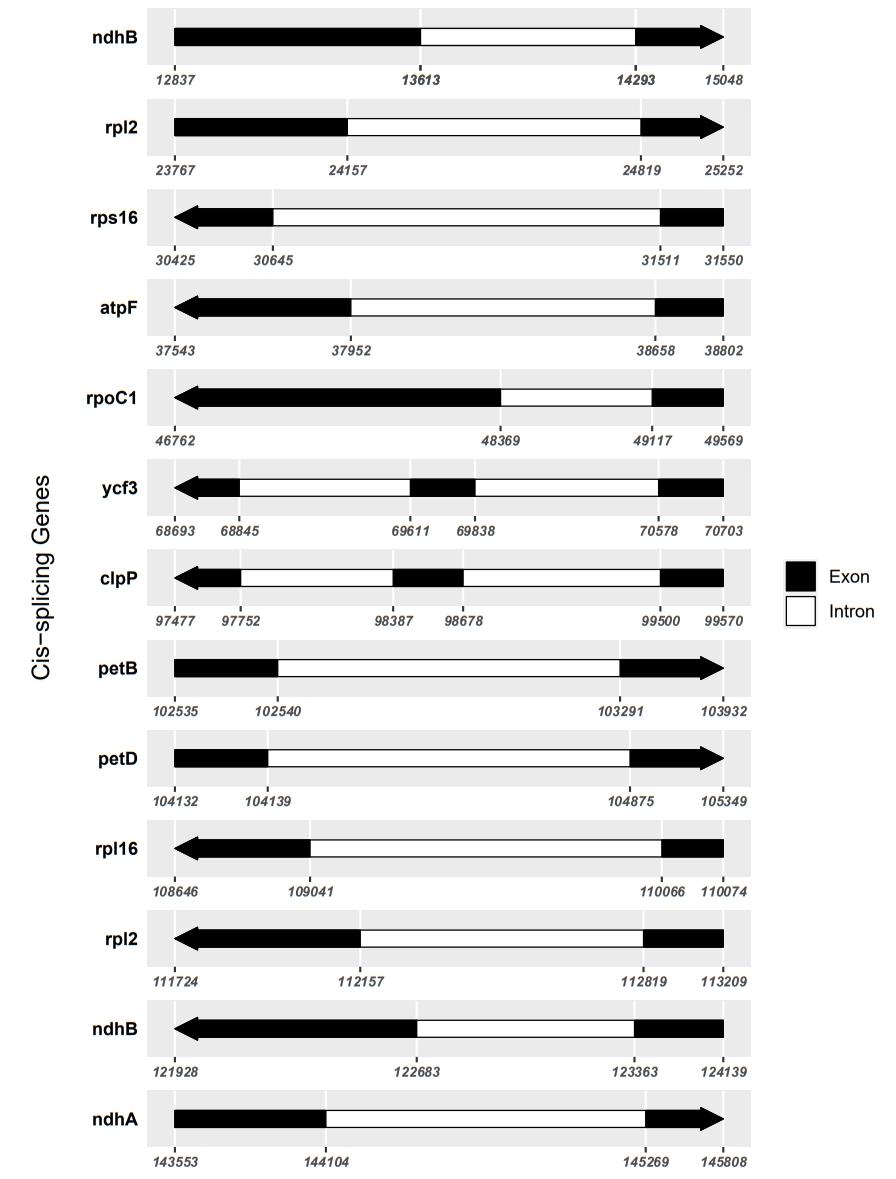


**Figure S2** Cis-splicing genes in chloroplast DNA of ‘Yunqie 9’.The map of the cis-splicing genes, including thirteen cis-splicing genes (*ndhB, rpl2, rps16, atpF, rpoC1, ycf3, clpP, petB, petD, rpl16, ndhA*),the *ndhB and rpl2* were duplicates, eleven of them have one intron and two exons, and two have two introns and three exons.

**
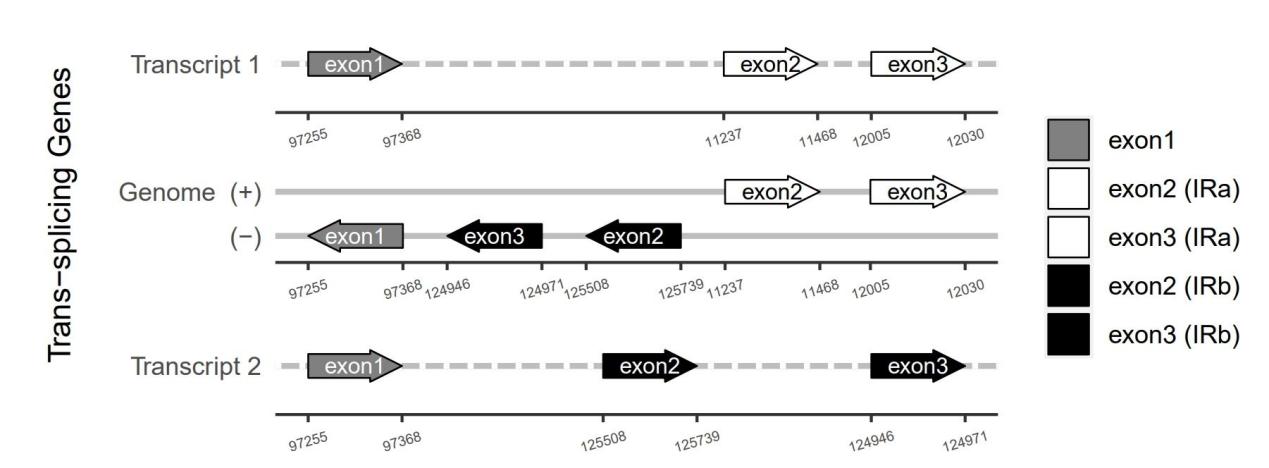
**

**Figure S3** Trans-splicing genes in chloroplast DNA of ‘Yunqie 9’
